# Identification and Management of the Hypervirulent Invasive *Klebsiella pneumoniae* Syndrome: A Unique and Distinct Clinical Entity

**DOI:** 10.1177/2324709618806552

**Published:** 2018-10-16

**Authors:** Mithu Maheswaranathan, Tue Ngo, Don C. Rockey

**Affiliations:** 1 Medical University of South Carolina, Charleston, SC, USA; 2 Carolinas Medical Center, Charlotte, NC, USA

**Keywords:** sepsis, abscess, meningitis, ventriculitis, diabetes mellitus

## Abstract

In this article, we describe the case of a 61-year-old woman who presented with altered mental status and was found to have *Klebsiella pneumoniae* meningitis, bacteremia, and hepatic abscess. Despite intravenous antibiotic therapy and percutaneous drainage of the hepatic abscess, the patient developed worsening meningitis with ventriculitis, which led to her death. This invasive *Klebsiella* syndrome is characterized by metastatic infections with a hypervirulent strain and a poor clinical prognosis. Important elements of this patient’s case—including the widespread and highly aggressive course—and of those reported in the literature are reviewed. Although the invasive *Klebsiella* syndrome is an increasingly recognized clinical entity, most often in the Eastern hemisphere, it remains rare in the United States with approximately 20 cases reported in the literature, and given its high morbidity, it is vitally important for clinicians to recognize.

## Introduction

*Klebsiella pneumoniae* causes nosocomial and community-acquired infections, including pneumonia, bacteremia, urinary tract infection, surgical wound infections, liver abscess, cholangitis, and peritonitis. *K pneumoniae* has also been increasingly recognized as a cause of community-acquired pyogenic liver abscess, in the absence of underlying hepatobiliary disease, especially in patients from Eastern countries (Singapore, Hong Kong, Korea, and Vietnam) and in those with underlying risk factors such as diabetes mellitus.^1^ Metastatic infection has also been associated with *K pneumoniae* liver abscess.

Pyogenic liver abscesses are typically treated with management of the underlying condition (ie, biliary obstruction), drainage, and antibiotics and typically have an excellent prognosis.^2^ However, the biology, and especially the natural history of *K pneumoniae* liver abscess and associated invasive syndrome is substantial, with mortality rates ranging from 3% to 42%.^3^ Here, we report on a patient with invasive *K pneumoniae* syndrome with pyogenic liver abscess associated with meningitis and ventriculitis that was fatal.

## Case Presentation

A 61-year-old Chinese female with no known past medical history presented to the hospital with altered mental status for 1 week with associated fever, neck pain, nausea, and vomiting. It was not possible to obtain history from her due to her altered mental status; her family denied complaints of abdominal pain, headache, visual changes, focal weakness, chest pain, or dyspnea. She had been waking up in the middle of the night to cook meals and clean her house, and was intermittently somnolent. The patient did not smoke, drink alcohol, or use illicit drugs. She was born in China and immigrated to the United States, where she resided for the past 30 years with no foreign travel during that time. She had not seen a physician in her adult life and took no medications. Given her altered mental status and concern about the stability of her airway, she was intubated in the emergency department.

On admission to the intensive care unit, the patient’s vital signs were as follows: temperature 36.5°C, heart rate 90 beats/min, respiratory rate 22 breaths/min, blood pressure 108/61 mm Hg, and oxygen saturation of 100% on an FiO_2_ (fraction of inspired oxygen) of 40%. Physical examination demonstrated a positive Brudzinski sign and neck stiffness, even while sedated. Pupils were equal round and reactive to light, and she responded to painful stimuli. Her lungs were clear to auscultation bilaterally, and cardiac examination was unremarkable without a murmur. Her abdominal examination was normal with no hepatomegaly or ascites.

Admission laboratory data demonstrated white blood cell count of 19 900/mm^3^ (81% neutrophils), hemoglobin 11.8 g/dL, platelet count 170 000/µL, creatinine 0.5 mg/dL, bilirubin 0.6 mg/dL, aspartate transaminase 49 IU/L, alanine transaminase 81 IU/L, alkaline phosphatase 142 IU/L, and albumin 1.9 g/dL. Electrolytes were within normal limits. C-reactive protein was 4.19 mg/dL, and erythrocyte sedimentation rate was >100 mm/h. She had mild hyperglycemia on admission, with glucose 132 mg/dL and hemoglobin A1c of 6.4%.

**Figure 1.**
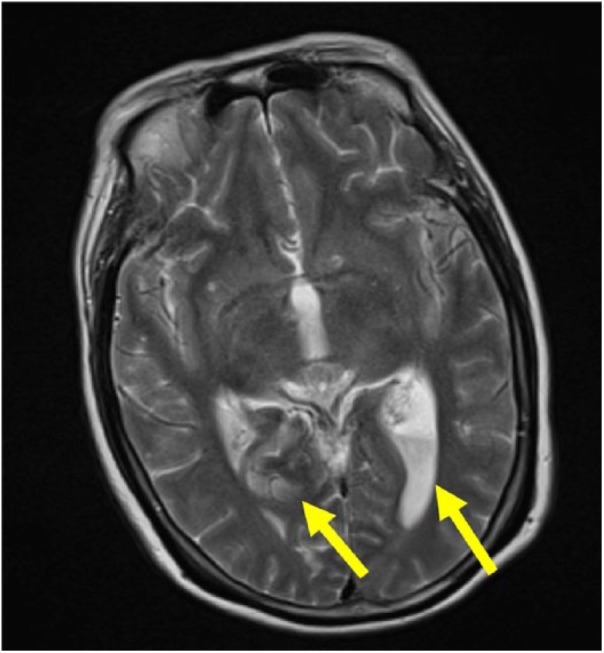
Brain magnetic resonance imaging demonstrating meningitis and ventriculitis. The arrow on the left points to diffuse leptomeningeal enhancement most conspicuous along the bilateral temporal lobes/insula, subarachnoid space of the basal cisterns and ventricular system. The arrow on the right points to additional proteinaceous material/pus within the subarachnoid space and dependently in the bilateral lateral ventricles.

Magnetic resonance imaging of the brain demonstrated diffuse leptomeningeal enhancement most conspicuous along the bilateral temporal lobes and insula, subarachnoid space of the basal cisterns, and the ventricular system, and proteinaceous material and pus were visualized within the subarachnoid space and bilateral lateral ventricles (Figure 1). Neurosurgery placed an external ventricular device to help drain cerebrospinal fluid (CSF). Lumbar puncture yielded clear CSF with an opening pressure of 22 cm H_2_O, a white blood cell count of 5051/mm^3^ (neutrophils 87%), protein 803 mg/dL, and a glucose 4 mg/dL. CSF culture was positive for *K pneumoniae*.

**Figure 2.**
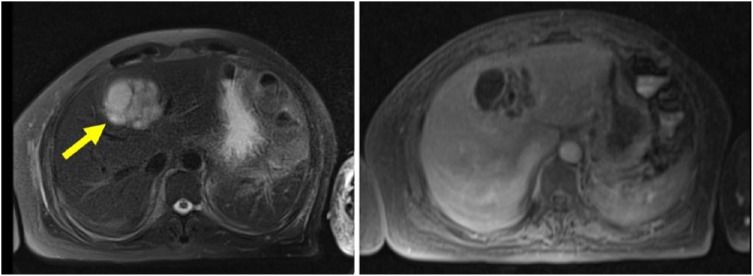
Liver magnetic resonance imaging demonstrating a pyogenic liver abscess. The arrow points to a thick-walled cystic lesion 6.3 × 4.6 cm with small amount of surrounding edema (the image on the left is a T1 image and right is a T2 image). There is no intrahepatic or extrahepatic biliary dilatation, lymphadenopathy, or free fluid. Aspiration of the complex multiloculated lesion returned purulent material consistent with pyogenic hepatic abscess.

Magnetic resonance imaging of the abdomen demonstrated a complex multiloculated cystic space occupying lesion 6.3 × 4.6 cm with a small amount of surrounding edema and no intrahepatic or extrahepatic biliary dilatation (Figure 2). Percutaneous aspiration yielded 25 cc of thick, purulent fluid, which was positive for *K pneumoniae*; a catheter was placed into the hepatic abscess. Blood cultures were positive for *K pneumoniae*.

She was initially treated with meropenem and intrathecal gentamicin. She subsequently developed seizures. With concern of lowered seizure threshold from meropenem, this was changed to cefepime. Ciprofloxacin was added as she developed worsened mental status, and repeat central nervous system (CNS) imaging revealed increasing leptomeningeal enhancement and fluid within the occipital horns, consistent with worsening meningitis and ventriculitis. Her external ventricular devices repeatedly clogged and a total of 6 external ventricular drains were placed. Thirty days after admission, she developed diabetes insipidus, uncal herniation, and progressed to brain death.

## Discussion

*Klebsiella pneumoniae* is recognized as a common pathogen in both nosocomial and community-acquired infections. It is a classic cause of pneumonia, especially in alcoholic and diabetic patients. It is also well known to cause bacteremia and pyogenic liver abscess. To date, some 800 cases of *K pneumoniae* liver abscess have been reported from Eastern countries and Southeast Asia, but a substantially lower number have been reported in Europe, North America, and South America.^1^ Several case series in the United States have evaluated pyogenic liver abscess, with those involving *K pneumoniae* being reported to occur in 7% to 35%.^4,5^ A very small proportion (approximately 20 cases overall) of those reported in the United States appear to have been caused by the hypervirulent strain.^6^ *K pneumoniae* infection has also become a major cause of community-acquired meningitis in Asia, and remarkably has been linked to pyogenic liver abscess.^3^ Particularly noteworthy are the points that invasive *K pneumoniae* syndrome is associated with primary hepatic abscess and systemic infection is associated with a high morbidity and mortality.

This hypervirulent strain of *K pneumoniae* was first reported in Taiwan starting in the mid-1980s, where patients without hepatobiliary disease presented with community-acquired hepatic abscess with a propensity for disseminated spread.^1,3,6^ This particular strain appears to be different from classic *K pneumoniae* strains, because it has a propensity for metastatic spread to unusual sites and because it often occurs in previously healthy hosts^3^; the metastatic spread and aggressive nature of the disease has led to the term “Invasive *Klebsiella* Syndrome.” It has been proposed that the syndrome is “definitive” in the presence of a *K pneumoniae* liver abscess with extrahepatic infection, including in the CNS, endophthalmitis, or necrotizing fasciitis.^1^ Our patient met the clinical criteria for the definitive invasive syndrome as evidenced by liver abscess and concomitant meningitis and ventriculitis.

Patients with primary *K pneumoniae* liver abscess have been reported to develop metastatic infection at a rate of 8% to 24%,^1,2^ with the most common metastatic foci being meningitis or endophthalmitis^1^ in the invasive syndrome. In addition to meningitis, other CNS infections including brain abscess and subdural empyema have been described in the metastatic syndrome.^3^ Other sites of systemic infection include the lungs (septic pulmonary emboli or empyema), musculoskeletal system (osteomyelitis, muscle abscess, or necrotizing fasciitis),^1^ or the spleen (splenic abscess) or the peritoneum.^3,7^ High mortality has been noted in patients with meningitis, septic pulmonary emboli, or empyema.^1,2^

The most common clinical manifestations in patients with liver abscesses are fever, chills, abdominal pain, nausea, and vomiting.^2^ Interestingly, abdominal pain and gastrointestinal symptoms are not always present in patients with the invasive form of the disease.^1^ Importantly, several risk factors have been put forward for the invasive syndrome, including Asian descent and diabetes mellitus.^1^ The invasive syndrome has been reported to be prominently associated with diabetes mellitus.^5^ Additionally, typical laboratory findings include leukocytosis, thrombocytopenia, hyperglycemia, abnormal liver function tests, and elevated C-reactive protein. Our patient was of Asian descent and had impaired glucose tolerance without meeting diagnostic criteria for diabetes mellitus.

Patients with the invasive syndrome have been found to have a hypermucoviscous *K pneumoniae* strain and also serotypes K1 or K2.^1^ This hypermucoviscous variant phenotype is identified by a positive “string test” in the laboratory. The positive “string test” is defined as an inoculation loop generating a viscous string greater than 5 mm in length when manipulating bacterial colonies on an agar plate.^1,3^ Our patient’s colonies were reported to exhibit a positive string test. It has been hypothesized that the hypermucoviscosity phenotype of the hypervirulent *K pneumoniae* strain may make catheter drainage difficult.^3^ In our patient, we hypothesize that the high viscosity of this organism likely explains the difficulty draining the hepatic abscess and the recurrent clogging of the external ventricular drains with need for repeated drain replacement.

The most effective antibiotic for treatment of *K pneumoniae* abscess includes the cephalosporins.^1^ Extended spectrum β-lactamase producing *K pneumoniae* are rare but can be treated with carbapenem therapy.^1,2^ The most appropriate duration of therapy ranges from a minimum of 2 to 4 weeks for solitary liver abscess to over 6 weeks for complicated (multiple) abscesses. In patients with diabetes mellitus, strict glycemic control appears to be important as this can prevent development of extrahepatic complications.^1^ Treatment of metastatic infection is typically guided by the site of foci involved.

Morbidity and mortality from the invasive *K pneumoniae* syndrome are substantial, with mortality rates ranging from 3% to 42%.^3^ *K pneumoniae* meningitis has a particularly high mortality, with rates from 38% to 91%.^8^ Thrombocytopenia has been associated with a 3-fold increased mortality rate over patients with normal platelet counts (37% vs 11%).^5^

The reason for the aggressive pathogenesis of the invasive *K pneumoniae* syndrome is unclear. Given the predilection for those of Asian descent, possibilities include genetically predisposed susceptibility or perhaps pathogen exposure based on geography. Studies have demonstrated that a substantial proportion of individuals of Asian descent are colonized with hypervirulent *K pneumoniae* strains, with the dominant site of colonization being the gastrointestinal tract.^3^ For example, studies of fecal carriers of *K pneumoniae* in healthy Chinese people have found a prevalence of *K pneumoniae* of 75% in healthy adults with high prevalence of the K1 or K2 serotype isolates (23%), which is considerably higher than prevalence in European fecal samples (10% to 19%).^1^ Thus, it is possible that colonization with the virulent K1 or K2 serotype may predispose to translocation of *K pneumoniae* in the setting of dysregulated gut barrier function from the gastrointestinal tract to the hepatic circulation.^1,2,9^

It is important for clinicians to be aware of the invasive *Klebsiella* syndrome, particularly with its increasing spread outside of Asia. Additionally, microbiologists should be aware that *K pneumoniae* with hypermucoviscous phenotype suggests an invasive *K pneumoniae* strain,^1^ which should prompt notification of clinicians to ensure appropriate screening for extrahepatic manifestations. Additionally, in any patient with *K pneumoniae* meningitis, endophthalmitis, or other extrahepatic infection (particularly in those of Asian descent or with uncontrolled diabetes mellitus), patients should be investigated for possible liver abscess. By recognizing the features of the invasive *Klebsiella* syndrome, rapid diagnosis and treatment may improve outcomes.

## Conclusion

The invasive *K pneumoniae* syndrome is associated with significant morbidity and mortality. Atypical Klebsiella infections associated with this invasive syndrome include meningitis, endophthalmitis, and necrotizing fasciitis. Additionally, in patients with atypical Klebsiella infection, a high index of suspicion for the presence of pyogenic liver abscess is important. Although the disseminated *K pneumoniae* has been previously reported in the literature, our case demonstrates the importance of recognizing disseminated *K pneumoniae* infection as part of an invasive syndrome with unique extrahepatic manifestations. Finally, this disease causes significant morbidity and mortality and appears to be underappreciated by clinicians in Western nations. Indeed, early recognition of the disorder is vitally important.
